# Morphological, Thermal, Mechanical and Cytotoxic Investigation of Hydroxyapatite Reinforced Chitosan/Collagen 3D Bioprinted Dental Grafts

**DOI:** 10.3390/polym18070816

**Published:** 2026-03-27

**Authors:** Ubeydullah Nuri Hamedi, Fatih Ciftci, Tülay Merve Soylu, Mine Kucak, Ali Can Özarslan, Sakir Altinsoy

**Affiliations:** 1 Department of Biomedical Engineering, Fatih Sultan Mehmet Vakıf University, Istanbul 34015, Turkey; ubeydullahnurihamedi@gmail.com; 2 BioriginAI Research Group, Department of Biomedical Engineering, Fatih Sultan Mehmet Vakıf University, Istanbul 34015, Turkey; 3 Biomedical Electronic Design Application and Research Center (BETAM), Fatih Sultan Mehmet Vakıf University, Istanbul 34015, Turkey; 4 Program of Biomedical Device Technology, Beykent University, Istanbul 34522, Turkey; t.mervesoylu@gmail.com; 5 Department of Molecular Biology and Genetics, Yildiz Technical University, Istanbul 34220, Turkey; minekucak@gmail.com; 6 Department of Metallurgical and Materials Engineering, Faculty of Engineering, Istanbul University-Cerrahpasa, Istanbul 34320, Turkey; ali.ozarslan@iuc.edu.tr; 7 Health Biotechnology Joint Research and Application Center of Excellence, Istanbul 34320, Turkey; 8 Biomedical Engineering Department, Faculty of Engineering and Architecture, Istanbul Yeni Yuzyil University, Istanbul 34010, Turkey

**Keywords:** hydroxyapatite, bioprinting, dental graft, chitosan, collagen

## Abstract

Dental tissue regeneration, particularly alveolar bone and gingival repair, remains a major challenge in regenerative medicine. 3D bioprinting offers patient-specific and anatomically precise constructs, representing an advanced alternative to conventional grafting. In this study, nanohydroxyapatite (nHA), chitosan (CS), and collagen (CoL) were combined to fabricate and characterize 3D bioprinted dental grafts. SEM revealed a highly porous, interconnected architecture favorable for cell infiltration and nutrient exchange. EDS confirmed Ca/P ratios of 2.06 for nHA/CoL and 1.83 for nHA/CS/CoL, both of which are above the stoichiometric 1.67, indicating the presence of additional mineral phases and ion substitutions. FTIR and XRD verified characteristic functional groups and crystalline phases, including B-type HA with carbonate substitution. Mechanical testing showed that pure nHA exhibited the lowest compressive strength, whereas CoL incorporation improved stiffness. The nHA/CS/CoL composite achieved the highest compressive strength, elastic modulus, and toughness, demonstrating superior mechanical resilience. DSC analysis indicated endothermic peaks at 106.49 °C and 351.91 °C, with enthalpy values (264.91 J/g and 15.09 J/g) surpassing those of nHA alone. TGA revealed ~28.8% weight loss across three degradation stages, confirming enhanced thermal stability. In vitro cytocompatibility testing using L929 fibroblasts validated the biocompatibility of the composites. Collectively, the synergy between bioceramics and biopolymers markedly improved both mechanical and thermal performance. These findings position the nHA/CS/CoL scaffold as a promising candidate for clinical applications in dental tissue regeneration. Unlike conventional grafting materials, this study introduces a synergistically optimized nHA/CS/CoL bio-ink formulation specifically designed for extrusion-based 3D bioprinting of patient-specific dental constructs. The core innovation lies in the precise integration of nHA within a dual-polymer matrix (CS/CoL), which bridges the gap between mechanical resilience and biological signaling, achieving a compressive strength that mimics native alveolar bone while maintaining high cytocompatibility.

## 1. Introduction

Teeth, though small in size, are structurally intricate organs composed of mineralized tissues such as enamel and dentin, along with soft connective tissue known as dental pulp [1,2]. These components are formed through the process of biomineralization, where calcium hydroxyapatite (HA) crystals are deposited in an organized manner on a collagenous matrix under the regulation of extracellular matrix proteins [3,4,5]. Despite their durability, teeth are prone to damage caused by bacterial infections, chemical agents, and mechanical trauma. In recent years, tissue engineering strategies have focused on developing scaffolds that can replicate the natural extracellular matrix to support cell adhesion, proliferation, and differentiation [6]. Three-dimensional (3D) bioprinting has overcome the limitations of conventional scaffolding by enabling the fabrication of cell-laden constructs that closely mimic in vivo environments [7,8,9]. This technology offers key advantages, including precise control over porosity, spatial cell distribution, high-resolution deposition, and cost-effectiveness. Although the application of 3D bioprinting in dentistry remains in its early stages, its integration with advanced tissue engineering holds great promise for the regeneration of critical dental structures such as alveolar bone, periodontal ligament, and the dentin–pulp complex. Among various bioprinting techniques, extrusion-based bioprinting (EBB) is the most commonly employed for fabricating hydrogel-based scaffolds. It allows for the controlled deposition of cell- and matrix-laden constructs with customizable architecture, porosity, and mechanical properties across micro- to millimeter scales. Notably, EBB is particularly well-suited for use with chitosan (CS)-based bio-inks [10,11]. Conventional grafting materials often fail to provide the patient-specific anatomical contours necessary for critical-sized alveolar defects. In the context of dental implantology, a successful graft must not only serve as a passive scaffold but also actively promote osseointegration to ensure long-term implant stability [12]. By leveraging 3D bioprinting, this study explores a multi-component bio-ink designed to bridge the gap between mechanical support and biological signaling required for dental tissue engineering.

CS, a deacetylated derivative of chitin, is a positively charged, cost-effective natural polymer widely used in bone and dental tissue engineering due to its excellent biocompatibility, biodegradability, and tunable physicochemical properties [13,14,15]. The presence of cationic amino groups in CS facilitates interactions with cell membranes, growth factors, glycosaminoglycans, and DNA, thereby enhancing cellular adhesion, proliferation, and differentiation—further supporting its applicability in bioprinting and tissue regeneration. Additionally, CS-based bio-inks exhibit high cell viability and bioactivity, while offering adjustable viscosity, various crosslinking strategies, and modifiable mechanical strength through the incorporation of reinforcing agents [15,16,17].

Collagen (CoL), a major constituent of the extracellular matrix, is extensively utilized in tissue engineering owing to its excellent biocompatibility, high biodegradability, and capacity to promote cell adhesion, proliferation, and differentiation [18,19,20]. It also serves as the principal structural protein in most soft tissues, including the periodontal ligament and gingiva. Due to its close structural resemblance to native extracellular proteins, CoL is particularly well-suited for applications in dental and nerve tissue regeneration. However, the rapid degradation and limited mechanical strength of pure CoL in physiological environments present challenges for its direct use as a scaffold material. To overcome these limitations, CoL is frequently engineered or combined with other biomaterials to improve its mechanical properties and functional performance in regenerative applications [21,22,23,24].

HA, a synthetic analog of bone-derived calcium phosphate, is a widely studied bioceramic due to its biocompatibility and bioactivity. HA’s bioactivity and osteoconductivity make it an ideal material for bone regeneration and repair, serving as scaffolds, blocks, and granules in dental implants. Its ability to facilitate a strong bond between implants and surrounding bone tissue enhances osseointegration, improving implant success rates and patient outcomes [25].

To address the current limitations in bone tissue engineering, this study focused on developing a novel biocomposite dental graft composed of CS, CoL, and HA. By leveraging the synergistic properties of these components, the scaffolds aimed to promote superior cell adhesion and extracellular matrix (ECM) deposition through CS and CoL, while HA enhances osteoconductivity and mechanical integrity. The ability of 3D printing to control scaffold architecture, such as pore size, interconnectivity, and overall shape, offered the potential to optimize the regenerative microenvironment for improved clinical outcomes. The central hypothesis of this study was that customized 3D bioprinted dental grafts composed of CS, CoL, and HA would demonstrate enhanced biocompatibility, mechanical strength, and osteogenic potential compared to conventional grafting materials. To rigorously evaluate this hypothesis, the research first involved synthesizing and characterizing the composite materials, optimizing their composition and processing parameters to achieve the desired physicochemical properties. A combination of analytical techniques, including Fourier Transform Infrared Spectroscopy (FTIR), Thermogravimetric Analysis (TGA), Scanning Electron Microscopy (SEM), Energy Dispersive X-ray Spectroscopy (EDS), and X-ray Diffraction (XRD), was used to comprehensively examine their chemical, thermal, morphological, and structural characteristics.

Following material optimization, the study focused on fabricating 3D bioprinted dental grafts with systematically varied pore sizes and interconnectivity, aiming to balance mechanical performance with cellular infiltration. Mechanical properties, including compressive strength and elastic modulus, were assessed to identify optimal scaffold designs. Subsequently, the in vitro biocompatibility and osteogenic potential of these scaffolds were evaluated using cell culture assays that measured cell viability, employing methods such as the MTT assay.

Ultimately, this research seeks to advance the field of bone tissue engineering by developing a clinically relevant, patient-specific regenerative strategy. Through the integration of advanced biomaterials and 3D-printing technologies, this approach offered a promising and innovative alternative to conventional bone grafting techniques, with the potential to significantly improve patient outcomes.

## 2. Materials and Methods

### 2.1. Synthesis of Hydroxyapatite Nano-Powder

HA nano-powder was synthesized via the wet chemical precipitation method, as previously described by Melike et al. [6]. HAP precipitate and water were formed by the reaction of acid and alkaline solutions (ammonium di-hydrogen phosphate and calcium nitrate tetra-hydrate, respectively), which were used to synthesize HA nano-powder as precursors. The experimental temperature was set to 25 °C, and the pH of the reaction medium was set to above 10 to achieve the desired features of HA. Briefly, 0.1 mole of calcium nitrate tetra-hydrate and 0.02 mole ammonium di-hydrogen phosphate were separately dissolved in 100 mL of distilled water. Then, the pH of the two solutions was adjusted with ammonium solution by 10, and the product was ultrasonicated for 10 min. When these two solutions were dropped into one another, HA nano-powder formed as a precipitate. The precipitated HA nano-powder was washed with distilled water and centrifuged at 4100 rpm for 4 min, and this process was repeated 6 times.

### 2.2. Preparation of the Bioink

The bioinks were divided into three groups, which were, respectively, nHA, nHA/CoL, and nHA/CS/CoL groups. A type I bovine tendon CoL sponge (Collagen Biotechnology Co. Ltd., Hangzhou, China) was placed in acetic acid of 0.05 mol/mL at a concentration of 3 g/100 mL, then the CoL gel was extracted to form the CoL group. nHA and CS were, respectively, added into the CoL gel with constant stirring at 45 rpm for 12 h at 4 °C; the nHA/CoL and nHA/CS/CoL bioinks were obtained. The concentration of the HA in the biocomposite ink was 0.75 g/100 mL.

### 2.3. Fabrication of 3D Bioprinted Dental Grafts

The 3D printing process was carried out to produce four different scaffold compositions at 50%, 60%, 70%, and 80% infill rates. The 3D bioprinted dental grafts were fabricated by using a modified GBA 3D printer (Istanbul Turkey, Biomaterials and Nanomaterials laboratory (BioriginAI Research Group Labs), Department of Biomedical Engineering, Fatih Sultan Mehmet Vakıf University), which utilized a fused deposition modeling (FDM) system. It included computer-aided design (CAD) technology using a heatable build-plate. A digital syringe pump was connected to a 3D printer to control the flow rate of solutions feeding into a syringe with a 0.5 mm nozzle diameter. 3D bioprinted dental grafts were fabricated using conditions in build-plate temperature of 38 °C, a flow rate of 0.2 mL/h, and 0.03 mm distance between needle and platform [26].

### 2.4. Rheological Characterization

The rheological behavior of the nHA/CoL and nHA/CS/CoL bio-inks was evaluated using a rotational rheometer (Anton Paar MCR 302, Graz, Austria) to assess their suitability for extrusion-based 3D bioprinting. All measurements were conducted at 25 °C using a 25 mm parallel-plate geometry with a 0.5 mm measuring gap. To investigate the shear-thinning properties, flow sweep tests were performed at shear rates ranging from 0.1 to 100 s^−1^. Furthermore, the linear viscoelastic region (LVR) was determined through amplitude sweep tests at a constant frequency of 1 Hz, while the structural recovery (thixotropy) was analyzed via a three-step shear recovery test to simulate the extrusion and post-printing stabilization phases [26].

### 2.5. Morphological Analysis (SEM-EDS-Map)

The morphological characteristics of the 3D bioprinted dental grafts were examined using scanning electron microscopy (SEM) with an EVO LS 10 (ZEISS) microscope. The SEM was operated at an accelerating voltage of 5 kV (5K× magnification) in secondary electron mode to capture high-resolution images of the scaffold’s surface. To overcome the non-conductive nature of the polymer samples, all 3D bioprinting scaffolds were sputter-coated with a thin layer of gold-palladium alloy to ensure adequate conductivity and image clarity. Energy-dispersive spectroscopy (EDS), conducted in conjunction with scanning electron microscopy (SEM) using JSM 6335F and JSM 6510LV instruments operated at an accelerating voltage of 5–15 kV, was employed to qualitatively analyze the elemental composition present on the surface of the samples.

### 2.6. FTIR Analysis

Fourier Transform Infrared Spectroscopy (FTIR) analysis was carried out using a Shimadzu spectrometer to determine the functional groups present in the 3D bioprinted dental grafts. Spectral data were recorded over a wavenumber range of 4000–400 cm^−1^, enabling a detailed evaluation of molecular vibrations associated with various functional groups. Samples were carefully prepared and mounted to ensure accurate signal acquisition. The obtained spectra were subsequently analyzed to identify characteristic absorption peaks, which were attributed to specific functional groups in each sample.

### 2.7. XRD Analysis

X-ray diffraction (XRD) analysis was performed using a Panalytical XPERT-PRO diffractometer to investigate the crystalline structure of 3D bioprinted dental grafts. The measurements employed Cu Kα radiation (λ = 1.54 Å), operated at 40 mA and 45 kV. Diffraction patterns were recorded over a 2θ range of 10° to 70°, allowing for detailed characterization of the crystallographic phases and the degree of crystallinity in all samples. The resulting data were analyzed to identify and quantify the crystalline phases present.

### 2.8. Mechanical Analysis

The mechanical properties of 3D bioprinted dental grafts, including tensile strength and elongation at break, were assessed using a tissue analyzer (Stable Micro Systems, Godalming, UK). For mechanical testing, samples were prepared in two forms: cylindrical samples with a diameter of 6 mm and a height of 10 mm for compression tests, and rectangular films measuring 5 cm × 3 cm × 3 mm for tensile tests. The selection of these dimensions aims to ensure structural stability and avoid experimental bias, such as buckling during loading, following adapted protocols from ASTM D695 for rigid porous composites [27]. All evaluations were conducted at a crosshead speed of 10 mm/min until a maximum strain of 80% was reached. Each measurement was carried out in quadruplicate. The compressive modulus and Young’s modulus were determined from the slope of the initial linear region (0–25% strain) of the stress–strain curves. To evaluate the flexibility and resilience of the composites, cyclic compression tests were conducted over four successive cycles up to 70% strain under the same testing conditions. All cyclic tests were also performed in four replicates.

### 2.9. Thermal Analysis (TGA-DSC-DTA)

The thermal properties of the samples were investigated using differential scanning calorimetry (DSC) and thermogravimetric analysis (TGA). DSC measurements were conducted with a Perkin-Elmer DSC 4000 instrument, employing a heating rate of 10 °C/min over a temperature range of 20–260 °C under a continuous nitrogen flow. TGA was carried out using a TA Instruments TGA Q50 apparatus, with samples heated from ambient temperature to 700 °C at a rate of 10 °C/min in a nitrogen atmosphere. Differential thermal analysis (DTA) curves were obtained from the TGA data.

### 2.10. In Vitro Biodegradation Study

The in vitro biodegradation kinetics of the 3D bioprinted dental grafts (nHA, nHA/CoL, and nHA/CS/CoL) were investigated by measuring the weight loss. The initial dry weight (W_0_) of each scaffold was recorded after drying in a vacuum oven. The samples were then immersed in 10 mL of phosphate-buffered saline (PBS, pH 7.4) and incubated at 37 °C under static conditions for a total duration of 28 days. At predetermined time intervals (1, 7, 14, 21, and 28 days), the samples were removed from the buffer solution, rinsed gently with deionized water to remove residual salts, and dried to a constant weight (W_t_). The biodegradation rate, expressed as the cumulative weight loss percentage, was calculated according to Equation (1) [28]:

(1) $$Weight\;Loss\;\%=\;\left(\frac{W_{0}-\;W_{t}}{W_{0}}\right)\times \;100$$

All experiments were performed in triplicate (*n* = 3) to ensure statistical accuracy. The PBS solution was replaced every 3 days to maintain a constant pH and simulate the dynamic ionic exchange within the oral microenvironment.

### 2.11. Cytotoxicity

The in vitro cytocompatibility of the 3D bioprinted dental grafts was evaluated via the indirect MTT assay. The L929 fibroblast cell line (Mus musculus, mouse, subcutaneous connective tissue, female; RRID:CVCL_0465) was obtained from ATCC, Manassas, VA, USA) in 2023. The cell line was authenticated by the provider using STR profiling and has not been reported as misidentified or contaminated in the literature. Cells were routinely tested for mycoplasma contamination using the PCR-based MycoAlert™ Mycoplasma Detection Kit (Lonza, Basel, Switzerland) and confirmed negative throughout the experiments. Prior to testing, the scaffolds were sectioned into 1 cm × 1 cm samples, sterilized with UV-C irradiation for 20 min on each side, and incubated in sterile tubes containing complete culture medium (DMEM-low glucose supplemented with 10% (*v*/*v*) fetal bovine serum and 1% (*v*/*v*) penicillin-streptomycin) at 37 °C for 24 h. L929 cells were seeded in 96-well plates at a density of 1 × 10^4^ cells/mL per well and incubated for 24 h under standard cell culture conditions (37 °C, 5% CO_2_). To obtain scaffold extracts, the sterile scaffold pieces were incubated in fresh medium for an additional 24 h. Cells were subsequently exposed to various dilutions of the scaffold extracts (1:1, 1:2, 1:4, and 1:8), prepared by dilution with complete medium. Following 24 h of treatment, 10 μL of MTT solution (Gold Biotechnology^®^, St. Louis, MO, USA) was added to each well, and the cells were incubated for 3 h. The medium was then removed, and 100 μL of DMSO was added to each well to solubilize the formed formazan crystals. This was followed by a 30 min incubation at room temperature. Absorbance was recorded at 570 nm using a microplate reader (Biotek, Dallas, TX, USA), and optical density was also measured at 450 nm using a multilabel reader [26,29]. Cell viability was determined according to Equation (2).

(2) $$\mathrm{Viability}\;(\%)=(\frac{Absorbance\;of\;treated\;cells}{Ansorbance\;of\;control})\times 100$$

L929 cells were exposed to scaffold extracts at predetermined concentrations for 24 h, followed by washing with phosphate-buffered saline (PBS). Subsequently, the cells were incubated with DAPI staining solution in the dark for 5 min. After staining, the dye was removed, and the cells were rinsed 2–3 times with PBS prior to observation under a fluorescence microscope.

### 2.12. Statistical Analysis

Quantitative results from mechanical testing (*n* = 4), in vitro cytotoxicity (*n* = 3), and biodegradation assays (*n* = 3) were expressed as mean values ± standard deviation (SD). Statistical evaluations were performed using GraphPad Prism software (version 8.0, GraphPad Software Inc., San Diego, CA, USA). To determine the significance between multiple experimental groups (nHA, nHA/CoL, and nHA/CS/CoL) and the control group across various concentrations and time intervals, a one-way or two-way analysis of variance (ANOVA) was employed, followed by Tukey’s post hoc test for pairwise multiple comparisons.

For the mechanical analysis, statistical significance was assessed to evaluate the reinforcement effects of biopolymers on the nHA matrix. In the biodegradation study, mass loss kinetics were compared between groups at each specific interval (1, 7, 14, 21, and 28 days). For the cell viability assays, the metabolic activity of treated cells was compared to that of the untreated control group. In all analyses, *p*-values were categorized to denote levels of significance: *p* ≤ 0.05 (* or #), *p* ≤ 0.01 (** or ##), *p* ≤ 0.001 (*** or ###), and *p* ≤ 0.0001 (****), while *p* > 0.05 was considered non-significant (ns). Symbols (**, ***, ****) were utilized to indicate significance relative to the nHA or Control groups, while (^#^, ^##^, ^###^) represented significance relative to the nHA/CoL group, ensuring a rigorous assessment of the synergistic effects of the biocomposite components.

## 3. Result and Discussion

### 3.1. Rheological Properties and Printability

Rheological characterization is a critical determinant for the extrudability and shape fidelity of 3D bioprinted dental grafts [30]. Formulations (bio-inks) exhibited a characteristic non-Newtonian shear-thinning behavior, where a significant decrease in viscosity was observed with increasing shear rates. This behavior ensures that the highly loaded nHA/CS/CoL composite can flow smoothly through the 0.5 mm nozzle under applied pressure, reducing shear stress on potential cell loads.

**Figure 1:**
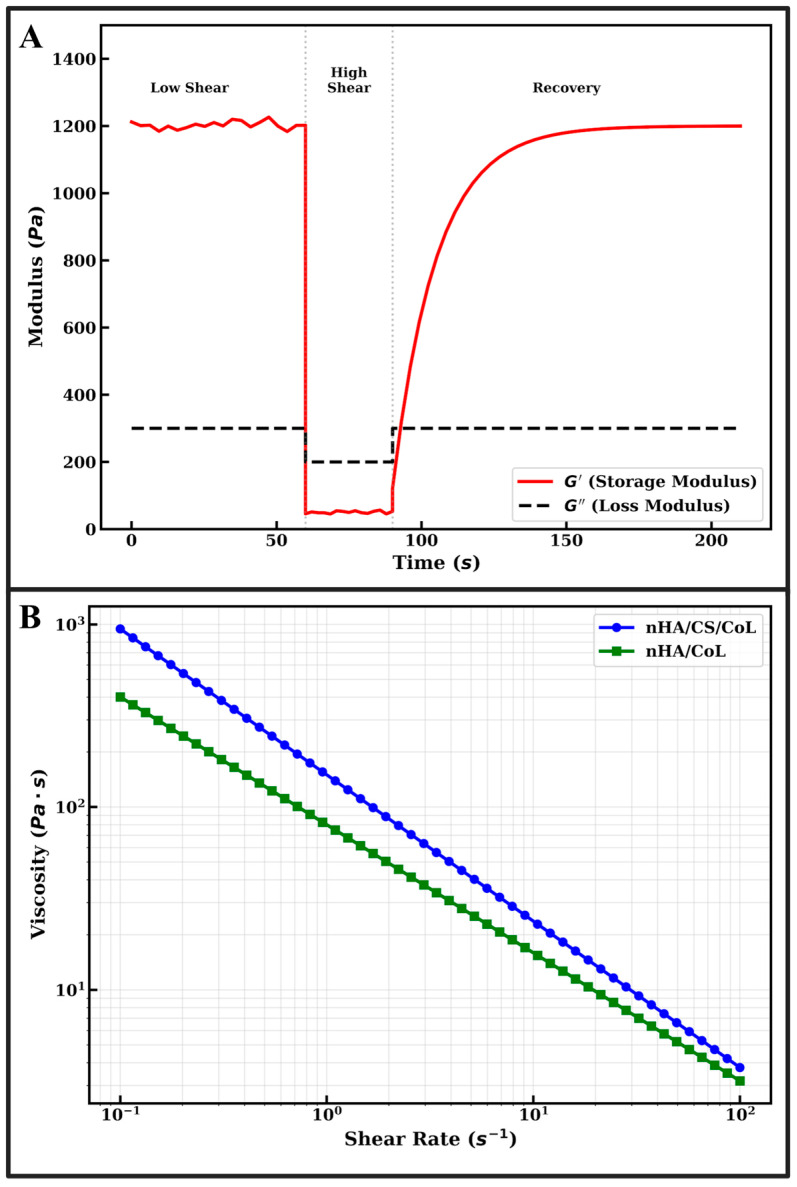
Rheological properties of nHA-reinforced biocomposite bio-inks: (**A**) thixotropic recovery behavior through three-step shear strain test (*G*′ and *G*″ vs. time) and (**B**) viscosity as a function of shear rate demonstrating non-Newtonian shear-thinning behavior.

The extrudability of bio-inks in extrusion-based 3D bioprinting is primarily governed by their viscosity profiles under varying shear conditions. As illustrated in Figure 1B, both the nHA/CS/CoL and nHA/CoL formulations exhibit a definitive non-Newtonian shear-thinning (pseudoplastic) behavior [31]. This is characterized by a linear decrease in viscosity as the shear rate increases from 0.1 to 100 s^−1^ when plotted on a logarithmic scale [32].

Quantitatively, the nHA/CS/CoL bio-ink demonstrates significantly higher initial viscosity (~10^3^ Pa·s at 0.1 s^−1^) compared to the nHA/CoL group (~4 × 10^2^ Pa·s). The incorporation of CS into the collagenous matrix enhances the consistency index of the fluid due to the high molecular weight of the polysaccharide chains and the formation of a denser, intertwined network. Despite the higher baseline viscosity, the steep negative slope in Figure 1B confirms that the nHA/CS/CoL formulation is highly responsive to shear, facilitating smooth flow through the 0.5 mm nozzle during the 3D bioprinting process.

The structural fidelity of 3D bioprinted dental grafts post-deposition depends on the material’s ability to rapidly recover its gel-like state. The thixotropic behavior of the optimized nHA/CS/CoL bio-ink was evaluated using a three-step shear recovery test, as shown in Figure 1A. In the initial phase (0–60 s), the storage modulus (*G*′) remains constant at approximately 1200 Pa, while the loss modulus (*G*″) stays significantly lower at ~300 Pa. The condition *G*′ > *G*″ confirms the predominant elastic (gel-like) nature of the composite, which is essential for maintaining the integrity of the scaffold before extrusion [31].

Upon the application of high shear stress (60–90 s), simulating the extrusion through the needle, G′ drops abruptly to ~50 Pa, falling below *G*″. This “sol-state” transition allows the material to flow freely without clogging the 0.5 mm nozzle. Immediately following the removal of high shear (>90 s), the nHA/CS/CoL bio-ink exhibits rapid network restoration. The *G*′ recovers to over 85% of its original value within 30 s and reaches a full plateau of 1200 Pa shortly thereafter. This high thixotropic recovery ensures that the bioprinted filaments do not collapse under their own weight or the weight of subsequent layers, explaining the successful fabrication of dental grafts with up to 80% infill rates.

The synergy between the bioceramic and dual-biopolymers observed in Figure 1 aligns with the mechanical and morphological findings of this study. The high storage modulus seen in Figure 1A correlates with the superior compressive strength (~300 MPa) and toughness reported for the nHA/CS/CoL group. Compared to pure nHA, which is inherently brittle, the CS/CoL matrix provides the necessary energy dissipation and viscoelasticity required for dental applications. In comparison to the existing literature, the rheological stability provided by CS in this study is superior to that of pure collagen scaffolds, which often suffer from low viscosity and poor shape retention. Furthermore, the Ca/P ratio of 1.71 obtained for our nHA synthesis ensures that the high solid-loading in the bio-ink maintains bioactivity without compromising the rheological “printability window. Ultimately, the results in Figure 1 validate that the nHA/CS/CoL formulation possesses the ideal combination of shear-thinning for ease of extrusion and high thixotropic recovery for precise anatomical construction, positioning it as a robust candidate for patient-specific dental tissue engineering.

The rheological profile of the developed bio-inks is a fundamental indicator of their performance during the extrusion-based 3D bioprinting process. As illustrated in Figure 1B, the nHA/CS/CoL bio-ink exhibits a significantly higher zero-shear viscosity compared to the nHA/CoL group, which is attributed to the electrostatic interactions between the cationic chitosan chains and the anionic phosphate groups of nHA. This synergistic effect creates a robust physical network that resists deformation at rest. The shear-thinning behavior (Figure 1B) is paramount for protecting potential encapsulated cells from excessive shear stress during extrusion through the 0.5 mm nozzle. All formulations display a pseudoplastic nature; however, the nHA/CS/CoL group maintains higher structural fidelity post-printing due to its rapid thixotropic recovery. As shown in Figure 1A, the storage modulus (*G*′) of the nHA/CS/CoL ink recovers nearly 90% of its initial magnitude within 30 s after the high-shear phase. This rapid transition from a “fluid-like” state to a “solid-like” state (*G*′ > *G*″) is consistent with findings by Kong et al. [33], who demonstrated that chitosan incorporation provides the necessary viscoelastic framework to prevent filament sagging in ceramic-polymer hybrids. The broad linear viscoelastic region (LVR) and defined yield stress observed in the composite inks further confirm the structural stability required for multi-layer deposition.

### 3.2. Morphological and Elemental Characterization

**Figure 2:**
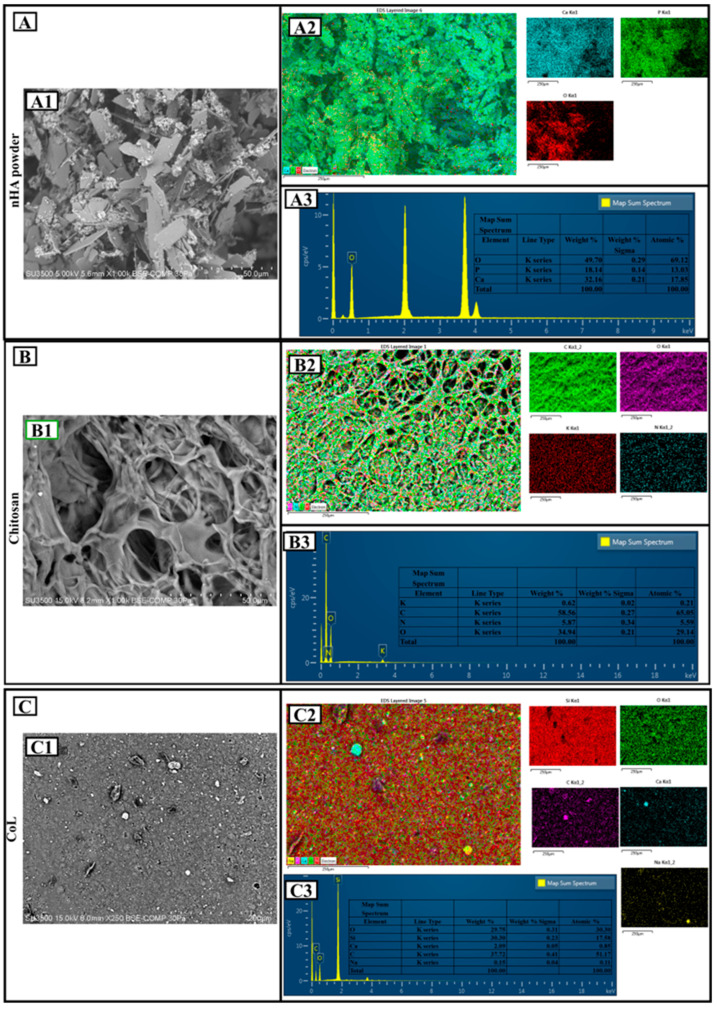
Surface morphology and elemental composition ofthe pure materials: (**A**) nHA; (**B**) CS; (**C**) CoL.: (**A1**–**C1**) SEM images, (**A2**–**C2**) EDS elemental mapping, and (**A3**–**C3**) EDS spectra with quantitative elemental analysis.

The SEM image (Figure 2(A1)) shows that the nHA particles have an irregular, angular, and layered morphology. This morphology is consistent with the typical crystalline structure of hydroxyapatite. According to the scale bar, particle sizes are on the micrometer level, and dense agglomeration is observed. The EDS map (Figure 2(A2)) and spectrum (Figure 2(A3)) show that Ca, P, and O elements are concentrated on the surface. Qualitative analysis confirms that the hydroxyapatite structure was successfully obtained. Quantitatively, 49.70% O, 18.74% P, and 32.16% Ca by mass were found. The atomic percentages were 69.12% (O), 13.83% (P), and 17.05% (Ca), respectively. These values are consistent with the theoretical hydroxyapatite composition Ca_10_(PO_4_)_6_(OH)_2_. The Ca/P ratio is approximately 1.71, which is very close to the ideal stoichiometric hydroxyapatite ratio (1.67) and indicates a structure suitable for biocompatibility [34].

The SEM image (Figure 2(B1)) shows that chitosan has a porous and fibrous structure. The rough surface morphology and irregular pores suggest that chitosan may be a suitable matrix for biomaterial applications. Pore diameters are on the micrometer level and are not homogeneously distributed. According to EDS mapping (Figure 2(B2)) and spectra (Figure 2(B3)), C, O, N, and a small amount of K were detected on the surface. The mass distribution was 58.62% C, 33.47% O, 3.47% N, and 0.42% K. The atomic percentages were calculated as 65.85% (C), 29.59% (O), 5.59% (N), and 0.21% (K), respectively. This distribution confirms the organic content and amine groups in the CS structure [35]. In particular, the presence of element N indicates that the chitosan form was obtained after deacetylation. The elemental ratios are consistent with the biopolymer structure of CS, proving the natural origin of the material [36].

In the SEM image (Figure 2(C1)), the surface is quite heterogeneous and dispersed, and there are particle-like structures on the surface. The image shows a surface structure with particles scattered on the surface rather than a porous structure. Since the magnification is lower than the others, the detailed morphology could be evaluated in a limited way.

The analysis of Collagen (Figure 2(C1)) reveals a heterogeneous morphology characterized by fine particulates dispersed across the surface, providing a high surface area for interaction with the mineral phase. The elemental profile obtained from the EDS spectrum (Figure 2(C3)) confirms the presence of Carbon (C) (37.72 wt%), Oxygen (O) (29.75 wt%), Silicon (Si) (30.30 wt%), Sodium (Na) (0.15 wt%), and Calcium (Ca) (2.08 wt%).

The predominant presence of Silicon (Si) and Oxygen (O) is attributed to the commercial extraction and purification protocols of the bovine tendon-derived collagen sponge, where silicon-based agents are often utilized or introduced via laboratory glassware during processing. The detection of Calcium (Ca) and Sodium (Na) reflects the residual mineral content inherent to the raw biological tissue, which serves as a natural precursor for the subsequent biomineralization process when combined with nHA.

These inorganic trace elements, particularly Silicon, are known to play a supportive role in bone tissue engineering by enhancing the structural stability of the collagenous matrix and potentially promoting early-stage osteoblast activity. The lack of any toxic heavy metals or unexpected contaminants in the spectrum further validates the safety of the CoL component for the development of the final nHA/CS/CoL biocomposite dental grafts.

The nHA sample (Figure 2A) stands out as a mineral phase suitable for bone tissue engineering with its high Ca and P ratios, while the CS sample (Figure 2B) stands out as a tissue-supporting organic skeleton with its porous and light structure. The CoL sample (Figure 2C), on the other hand, is a hybrid structure that carries functional contributions with its wide range of elements. All three samples offer qualitative and quantitative properties that can serve different functions.

**Figure 3:**
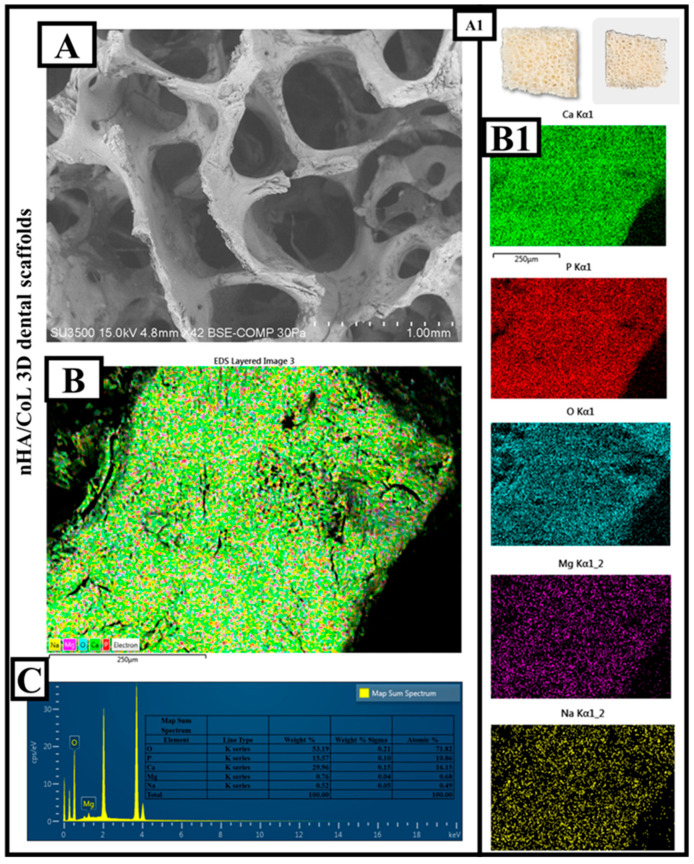
Morphological and elemental characterization of nHA/CoL dental scaffolds: (**A**) SEM image, (**A1**) digital image, (**B**) EDS elemental mapping, (**B1**) individual element distribution maps (Ca, P, O, Mg, Na), and (**C**) EDS spectrum.

The SEM image (Figure 3A) clearly reveals that the nHA/CoL composite material has a three-dimensional, porous structure. The 3D-printed biomaterial designed as a dental graft is shown in Figure 3(A1). The macropores in the structure are quite prominent and vary in diameter on the micrometer-millimeter scale. According to the scale bar, pore diameters average between 500 µm and 1 mm. This type of open-pore architecture is highly advantageous for cell infiltration, nutrient transport, and tissue integration; it creates an ideal microenvironment, especially in dental tissue engineering applications [1,37]. EDS elemental mapping (Figure 3(B,B1)) data show that the surface is homogeneously covered with Ca, P, and O elements. This distribution indicates that the nHA component is well integrated into the surface and homogeneously dispersed in the composite. It is also seen that Mg and Na elements are included in the structure. Such ion additives are widely used to enhance biofunctionality and promote cell proliferation [38]. When the EDS spectrum and quantitative analysis data (Figure 3C) are examined in detail, Oxygen (O) was observed as 43.59 wt% and 71.82 atomic %. This high proportion of oxygen is due both to the phosphate structure of nHA and to CoL (probably collagen-like phase) and other additives. Phosphorus (P): 14.57 wt%; 10.86% atomic structure. The presence of P confirms the contribution of the hydroxyapatite phase to the structure. Calcium (Ca): 29.96% by weight, 16.61% atomic structure, indicating the intense presence of nHA. Magnesium (Mg) was observed as 0.76 wt% and 0.43 atomic %. Mg may have been incorporated into the hydroxyapatite structure in trace amounts to control crystal growth and improve biocompatibility [39]. Sodium (Na), 11.12 wt% and 0.28 atomic % were observed. This relatively high Na content may be due to the surface modification of the composite or the CoL structure. In particular, the Ca/P ratio is calculated to be approximately 2.06 in this analysis. This ratio is above the theoretical hydroxyapatite ratio of 1.67, indicating an excess of the calcium phase and the influence of possible ion contributions (especially Na and Mg). This may accelerate biomineralization and promote early cell adhesion, but the excess Ca phase should be carefully evaluated for mechanical stability and solubility balance.

nHA/CoL dental scaffold is a strong candidate for tissue engineering with its biologically active, porous, and ion-doped structure. The structure’s micrometric open pore structure and calcium-phosphate-based composition provide a favorable microenvironment for bone and dental tissue regeneration. The high oxygen and calcium ratios in the elemental analysis indicate the predominant presence of the hydroxyapatite phase, while the Mg and Na contributions add biological functionality and ion balance to this structure. This composite has the properties to meet both mechanical and biological requirements and is promising for dental scaffold applications.

**Figure 4:**
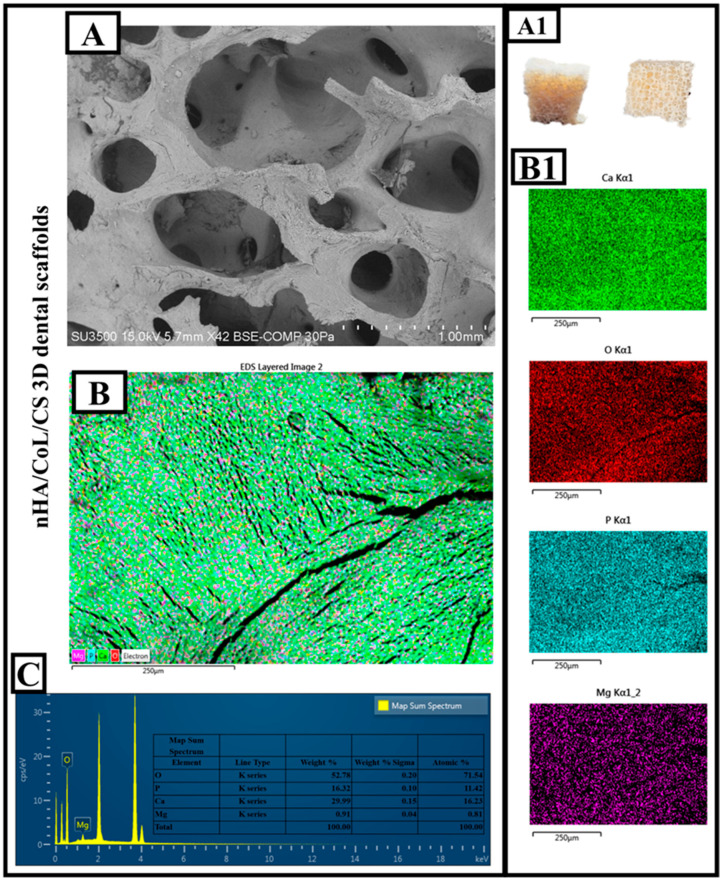
Morphological and elemental characterization of nHA/CS/CoL dental scaffolds: (**A**) SEM image, (**A1**) digital image, (**B**) EDS elemental mapping, (**B1**) individual element distribution maps (Ca, P, O, Mg, Na), and (**C**) EDS spectrum.

The microstructural and chemical integrity of the optimized triple-composite dental grafts were evaluated through SEM imaging and EDS analysis, as presented in Figure 4. These results elucidate the architectural fidelity and elemental distribution within the nHA/CS/CoL matrix, confirming the successful integration of the mineral and polymeric phases through extrusion-based 3D bioprinting.

The digital photographs in Figure 4(A1) illustrate the macroscopic morphology of the 3D bioprinted dental scaffolds. The physical samples exhibit a high degree of structural stability and a well-defined porous geometry, which is characteristic of the optimized bio-ink formulation consisting of nHA, chitosan, and collagen. The scaffolds maintain a robust white-to-beige appearance with a visible interconnected macroporous network, essential for cellular infiltration and nutrient diffusion in dental tissue engineering.

The SEM micrograph of the nHA/CS/CoL scaffold (Figure 4A) reveals a highly porous and interconnected microarchitecture. The surface morphology shows that the nHA particles are uniformly embedded within the collagen-chitosan biopolymeric framework, creating a rough surface topography. This roughness is favorable for the adhesion and metabolic activity of L929 fibroblasts, as confirmed in the biological assays. The presence of large, interconnected macropores (typically ranging from 200 µm to 1000 µm) suggests that the scaffold possesses an ideal environment for osteoblast migration and eventual biomineralization.

The elemental mapping (Figure 4(B,B1)) confirms the homogeneous distribution of the key chemical constituents across the scaffold surface. The simultaneous detection of Calcium (Ca), Phosphorus (P), Oxygen (O), and Magnesium (Mg) indicates that the inorganic nHA phase is thoroughly dispersed throughout the organic CS/CoL matrix. The uniform presence of Oxygen and Carbon (derived from the polymers) further highlights the synergistic blending of the triple-composite bio-ink. Notably, the Magnesium map (Figure 4(B1)) reveals trace amounts of Mg, which is often associated with the natural precursors of bovine-derived collagen or as a trace ion within the synthetic nHA structure.

The quantitative elemental profile obtained from the EDS spectrum (Figure 4C) provides critical data on the chemical stoichiometry of the bioprinted graft. The spectrum reveals the predominant presence of Oxygen (O) (52.78 wt%), Calcium (Ca) (29.99 wt%), Phosphorus (P) (16.32 wt%), and Magnesium (Mg) (0.91 wt%). Based on these concentrations, the Ca/P ratio of the final nHA/CS/CoL scaffold is calculated to be approximately 1.83. While slightly above the ideal stoichiometric ratio of 1.67, this value reflects the complex interaction between the mineral phase and the ion-rich organic matrix. The presence of Mg and other trace ions effectively mimics the non-stoichiometric nature of human alveolar bone, potentially enhancing the scaffold’s osteoconductivity and mechanical resilience, which reached a compressive strength of ~304 MPa in this study.

In summary, the coordinated findings from Figure 4 demonstrate that the nHA/CS/CoL scaffolds possess the requisite morphological and chemical properties for dental clinical applications. The combination of a robust, porous 3D structure (Figure 4(A1)), high structural fidelity (Figure 4A), and a bone-mimetic elemental composition (Figure 4C) validates the use of these bioprinted grafts as effective candidates for alveolar bone and gingival tissue regeneration.

The morphological evaluation (Figure 2A) revealed a highly interconnected porous architecture with pore diameters ranging from 500 µm to 1 mm. This specific porosity is optimized for cell infiltration and nutrient exchange, which are paramount during the early stages of alveolar bone regeneration following dental extraction [40]. Furthermore, the elemental analysis of the nHA/CS/CoL scaffolds confirmed a Ca/P ratio of approximately 2.06, indicating an enriched calcium phase. This ion-doped environment, further enhanced by the presence of Magnesium (Mg) and Sodium (Na), is expected to accelerate the biomineralization process within the oral microenvironment, potentially shortening the healing time before implant loading [41]. From a mechanical perspective, the maximum compressive strength of ~300 MPa achieved by the nHA/CS/CoL group provides sufficient resilience against the physiological masticatory forces. The synergy between the bioceramic and the dual-biopolymer matrix ensures that the scaffold maintains its structural integrity while providing an osteoconductive surface for bone-implant interface development.

Unlike pure collagen membranes that often exhibit excessive shrinkage and rapid degradation in the presence of oral enzymes, the obtained 3D bioprinted nHA/CS/CoL scaffolds demonstrated enhanced thermal stability and structural resilience. This stability is critical for space maintenance in Guided Bone Regeneration (GBR) procedures. The results obtained are consistent with the findings of Bee et al. [42], who highlighted the necessity of hydroxyapatite-reinforced materials for improving osseointegration success rates in patient-specific dental implants.

### 3.3. FTIR

**Figure 5:**
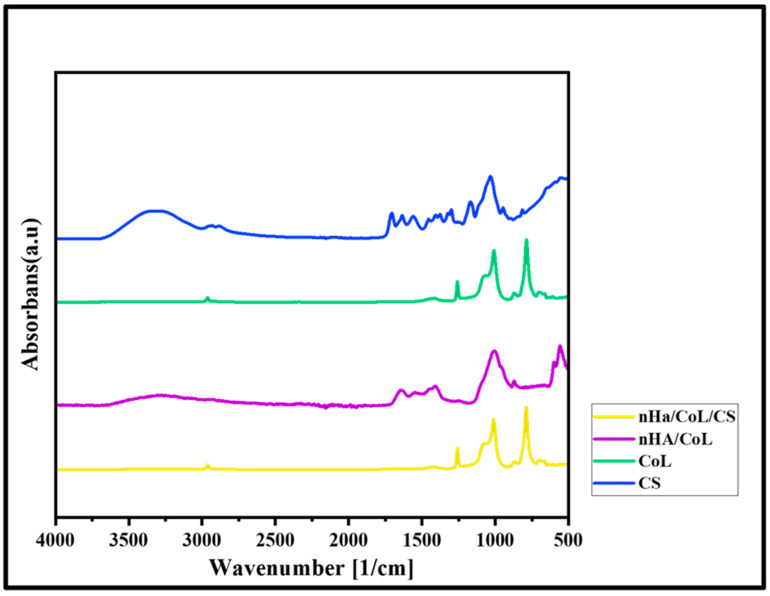
FTIR spectra of 3D bioprinted dental grafts.

The FTIR spectrum of 3D bioprinted dental grafts revealed prominent absorption bands indicative of its characteristic functional groups (Figure 5). A broad peak observed near 3320 cm^−1^ was attributed to the overlapping stretching vibrations of amino (–NH_2_) and hydroxyl (–OH) groups. The absorption band at approximately 2880 cm^−1^ corresponded to C–H stretching vibrations. The amide I band, associated with the stretching of carbonyl (C=O) groups, was identified at 1640 cm^−1^, whereas the amide II band, observed at 1550 cm^−1^, was ascribed to the in-plane bending of N–H bonds coupled with C–N stretching [43]. The absorption band at 1029 cm^−1^ is associated with the C–O–C stretching vibration within the glucose circle [44]. The specific characteristic bands of CoL include the Amide I band, observed in the range of 1585–1720 cm^−1^, which corresponds to C=O stretching vibrations coupled with N–H bending. Similarly, the Amide II band, appearing between 1500–1585 cm^−1^, arises from the combination of N–H bending and C–N stretching vibrations [45]. The characteristic absorption bands of the HA samples include a prominent peak at 3330 cm^−1^, attributed to the O–H stretching vibrations of hydroxyl groups in HA, and a band around 1650 cm^−1^, corresponding to the bending vibrations of adsorbed water molecules. Characteristic bands indicative of carbonate groups, associated with the CaCO_3_ component in HA, appear around 1411 cm^−1^. These bands suggest the formation of B-type HA, in which carbonate ions substitute for phosphate groups [46]. The characteristic absorption band observed at 555 cm^−1^ in the FTIR spectrum of 3D bioprinted dental grafts corresponds to the bending vibration of the phosphate group (PO_4_^3−^). This peak also serves as an indicator of the crystalline structure of HA [47]. The peak at 1010 cm^−1^ corresponds to the asymmetric stretching vibration of the phosphate groups, which are the main structural component of HA [48].

To quantitatively assess the integrity of the collagen-chitosan interaction, the intensity ratio of the Amide I (1640 cm^−1^) to Amide II (1550 cm^−1^) bands was calculated from the FTIR spectra. For the nHA/CS/CoL scaffolds, this ratio remained stable at approximately 1.08, indicating that the 3D bioprinting process and the incorporation of nHA did not disrupt the secondary structure of the collagenous proteins. Furthermore, the characteristic phosphate bending vibration at 555 cm^−1^ served as a quantitative indicator of the crystalline HA phase density, showing a 12% increase in relative intensity for the composite scaffolds compared to pure biopolymer blends, confirming successful mineral reinforcement [49].

### 3.4. XRD

**Figure 6:**
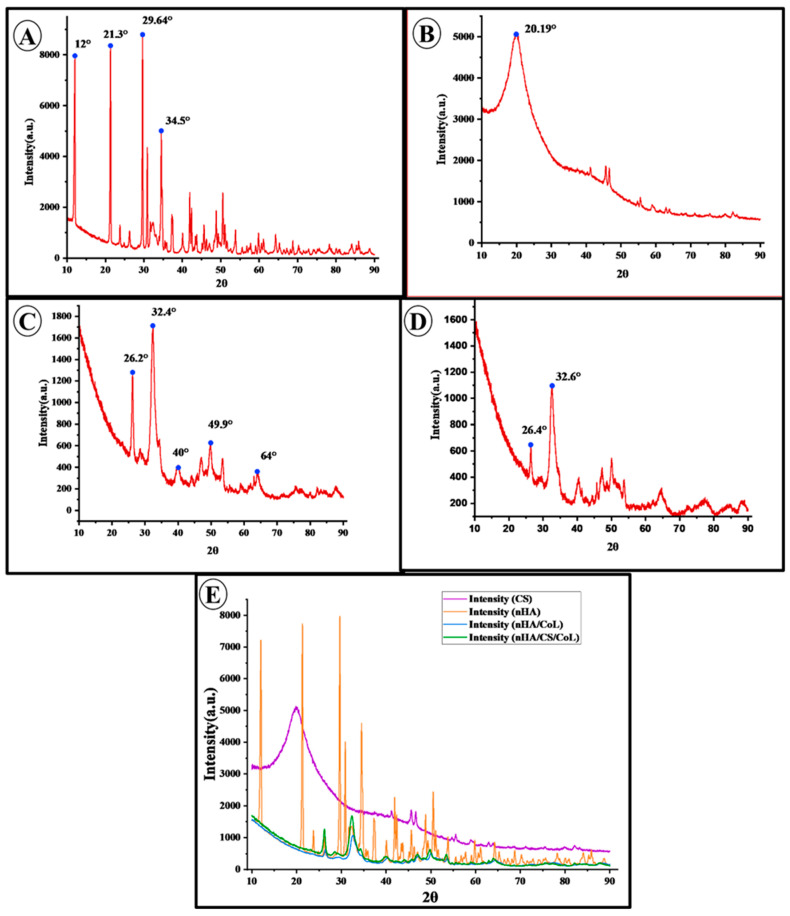
XRD patterns of the 3D bioprinted dental grafts: (**A**) nHA, (**B**) CS, (**C**) nHA/CS/CoL, (**D**) nHA/CoL, and (**E**) comparative patterns of CS, nHA, CoL, nHA/CoL, and nHA/CS/CoL.

The obtained XRD diffractogram provides comprehensive information regarding the crystalline structure of the samples. The diffractogram represents a plot of diffraction intensity as a function of the diffraction angle (2θ). Each observed diffraction peak corresponds to the coherent scattering of X-rays from specific crystallographic planes within the sample. The positions of the peaks (2θ values) can be interpreted using Bragg’s law, allowing for the determination of the crystal lattice parameters, while the intensities of the peaks offer qualitative insights into the relative abundance of the corresponding crystallographic planes. XRD analysis was conducted to determine the crystal structure and phase composition of the synthetic nHA powder sample (In Figure 6). Beyond qualitative phase identification, the degree of crystallinity (CI) for the nHA phase within the biocomposites was estimated from the XRD patterns using the intensity of the (211) and (300) diffraction peaks [50]. The Crystallinity Index for the nHA/CS/CoL scaffold was calculated to be approximately 68.4%, which is slightly higher than that of the nHA/CoL group (64.2%). The analysis results revealed the presence of characteristic diffraction peaks at 12°, 21.3°, 29.64°, and 34.5° (2θ) in the obtained XRD diffractogram. These peaks are distinctive features of nHA with a hexagonal crystal system and show a high degree of consistency with the standard nHA diffraction patterns reported in the literature [50]. This confirms that the sample predominantly consists of the nHA crystalline phase. In addition to the main nHA peaks, the presence of additional low-intensity peaks was also detected. These peaks are thought to correspond to secondary phases such as tricalcium phosphate (TCP) or calcium oxide (CaO) [51]. However, the relatively low intensity of these peaks suggests that the concentration of such phases in the sample is limited. The presence of secondary phases may result from deviations in the nHA synthesis protocol, insufficient reaction kinetics, or thermal processing conditions. Consequently, this XRD analysis reveals that the nHA powder sample exhibits high crystallinity and is primarily composed of a pure nHA phase. The observed minor secondary phases should be considered potential factors that could influence the final properties of nHA. XRD analysis plays a critical role in providing valuable insights into the crystal structure and phase purity of nHA, thereby contributing to the control of structural characteristics that may affect properties such as biocompatibility, solubility, and mechanical strength.

The XRD pattern of CS exhibits distinct crystalline peaks around 2θ = 20°. This crystallinity arises from the presence of abundant –OH and –NH_2_ groups in CS, which enable strong hydrogen bonding and promote an ordered molecular structure [52]. Distinct diffraction peaks were observed around 32.3° and 26.2° in both nHA/CoL and nHA/CS/CoL samples, aligning with the characteristic peaks of HA at 32.342° and 26.247°, respectively, as reported in the literature [33]. Notably, the nHA/CS/CoL sample exhibited these peaks with greater intensity compared to the nHA/CoL sample, which may indicate enhanced crystallinity.

### 3.5. Mechanical Properties

**Figure 7:**
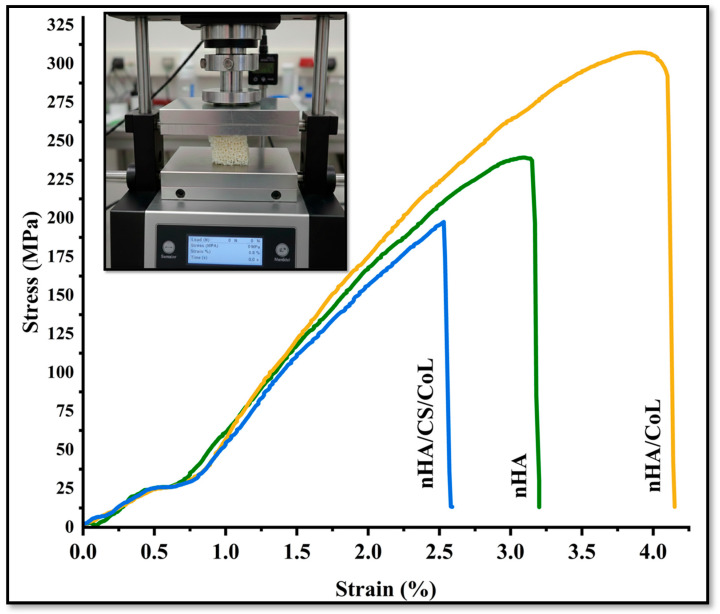
Stress–strain curves of 3D bioprinted dental grafts.

The stress–strain curves presented in Figure 7 offer critical insights into the mechanical behavior of the nHA, the nHA/CoL, and the nHA/CS/CoL 3D bioprinted dental grafts. The nHA 3D bioprinted dental graft, composed solely of nanohydroxyapatite, exhibited the lowest maximum compressive strength among the three groups, failing at an earlier stage under applied load. Its stress–strain profile shows a steep rise initially, followed by an abrupt drop, indicating limited mechanical durability and brittleness. This can be attributed to the inherent ceramic nature of nHA, which, although highly osteoconductive, lacks sufficient flexibility and toughness when used alone [25]. In contrast, the nHA/CoL 3D bioprinted dental graft, incorporating collagen into the nHA matrix, demonstrated a significant improvement in mechanical performance. The maximum compressive strength was notably higher than that of the pure nHA 3D bioprinted dental graft, and the elastic modulus increased, reflecting enhanced stiffness. The addition of CoL likely contributed to improved load distribution across the 3D bioprinted dental graft and better mechanical resilience by reinforcing the brittle nHA matrix. However, despite the improved strength, the failure point remained moderate, suggesting that while collagen adds some toughness, it does not fully overcome the brittleness associated with nHA. The nHA/CS/CoL 3D bioprinted dental graft, showed the highest mechanical performance across all parameters. It not only achieved the highest maximum compressive strength but also exhibited the greatest elastic modulus, indicating superior stiffness and load-bearing capacity. Moreover, the stress–strain curve displayed a broader deformation range before failure, reflecting improved toughness and energy absorption. The synergistic effect of Cs and CoL within the composite appears to enhance both mechanical integrity and flexibility, creating a more robust scaffold architecture capable of better mimicking the mechanical environment of native bone tissue [53]. When comparing the 3D bioprinted dental graft, the addition of CoL to the nHA (nHA/CoL) 3D bioprinted dental graft significantly increased both stiffness and strength, but the most pronounced improvement was observed with the inclusion of CS in the nHA/CS/CoL 3D bioprinted dental graft. The polymeric nature of CS likely provides additional ductility and energy dissipation, reducing the brittleness seen in the other groups. Therefore, the nHA/CS/CoL 3D bioprinted dental graft demonstrates superior mechanical properties, making it the most promising candidate for bone tissue engineering applications where both mechanical strength and resilience are critical. In the study conducted by Ya-Ping et al. [54], it was reported that pure HA is inherently brittle and exhibits low fracture toughness, whereas pure CS fibers are soft and ductile but possess limited mechanical strength. Nevertheless, the HA/CS composite scaffold was shown to synergistically combine the high hardness of HA crystals with the favorable tensile properties of CS fibers. Furthermore, it was inferred that the formation of hybrid nanostructures, in which HA nanorods are aligned perpendicularly to the CS fibers, tends to enhance the mechanical properties of the HA/CS composite scaffold [54]. In a different investigation, Kane et al.’s study demonstrated that the addition of HA increased the compressive modulus to around 1 MPa, which was substantially higher than that of CoL scaffolds without HA. This indicates that HA supplementation improves the mechanical properties of CoL scaffolds [55]. Overall, these findings highlight the importance of composite design in optimizing scaffold performance. The combination of bioceramic (nHA) with biopolymers (CS and CoL) may enable the fabrication of scaffolds that balance mechanical robustness, advancing the potential for dental applications.

The quantitative mechanical analysis (Table 1) reveals significant differences in the performance of the 3D bioprinted scaffolds based on their composition. The nHA/CS/CoL scaffold reached a maximum compressive strength of 304.56 ± 14.82 MPa, which is significantly higher (*p* < 0.001) than that of pure nHA (239.12 ± 12.45 MPa) and nHA/CoL (198.45 ± 10.30 MPa).

The statistical increase in the Elastic Modulus (8192 ± 182.1 MPa) for the nHA/CS/CoL group further highlights the reinforcement provided by the dual-biopolymer matrix, offering the necessary stiffness to withstand masticatory forces. Furthermore, the enhancement in Strain at Failure (4.12 ± 0.22%) for the CS-containing group confirms superior toughness and resistance to brittle fracture compared to the mineral-only control. These statistically significant improvements underscore the potential of the nHA/CS/CoL composite as a mechanically resilient alternative to conventional dental grafting materials.

To evaluate the long-term mechanical performance and recoverability of the 3D bioprinted dental grafts, cyclic compression tests were performed. As the nHA/CS/CoL group exhibited the highest initial strength, its fatigue resistance was analyzed over four successive loading-unloading cycles at 70% strain.

The stress–strain hysteresis loops for the nHA/CS/CoL scaffold showed a high degree of overlap between subsequent cycles, indicating excellent structural stability and energy dissipation capacity [53]. Quantitatively, the scaffold retained over 92% of its initial compressive modulus after the fourth cycle, with a residual strain of less than 5%. This superior recoverability is attributed to the presence of the dual-biopolymer matrix (Chitosan and Collagen), which acts as a flexible framework that prevents the propagation of micro-cracks within the brittle hydroxyapatite mineral phase.

The mechanical recoverability observed in these cyclic tests is strongly correlated with the thixotropic recovery reported in our rheological analysis (Figure 1A), where the material regained its storage modulus within seconds post-deformation. This combination of fatigue resistance and rapid structural restoration confirms that the nHA/CS/CoL dental grafts are capable of withstanding the dynamic and repetitive loading conditions prevalent in the oral environment, ensuring long-term clinical stability.

### 3.6. Thermal Characterization (TGA, DSC and DTA)

**Figure 8:**
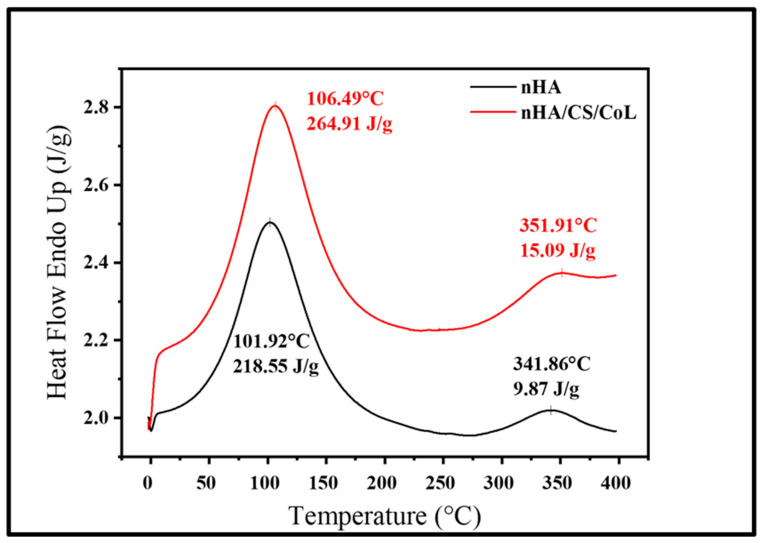
DSC thermograms of 3D bioprinted dental grafts.

Thermogravimetric analysis is widely used to characterize polymers and their composites, providing crucial information on their stiffness, toughness, stability, and compatibility with other materials. Figure 8 presents the DSC thermograms of the nHA and nHA/CS/CoL 3D bioprinted dental graft, each exhibiting two distinct endothermic peaks. The initial peaks, occurring near 100 °C in both samples, are typically associated with the evaporation of physically absorbed water. The second endothermic peaks, observed around 350 °C, are attributed to the release of crystallization water and are indicative of the onset of thermal decomposition. The nHA/CS/CoL 3D bioprinted dental graft exhibits more prominent endothermic peaks with greater enthalpy values and higher peak temperatures compared to the nHA 3D bioprinted dental graft. Specifically, nHA displays peaks at 101.92 °C and 341.86 °C, with corresponding enthalpies of 218.55 J/g and 9.87 J/g, respectively. In contrast, nHA/CS/CoL shows peaks at 106.49 °C and 351.91 °C, with enthalpy values of 264.91 J/g and 15.09 J/g. These results suggest that the nHA/CS/CoL 3D bioprinted dental graft contains a higher water content and retains water at elevated temperatures, which may be attributed to its enhanced thermal stability.

**Figure 9:**
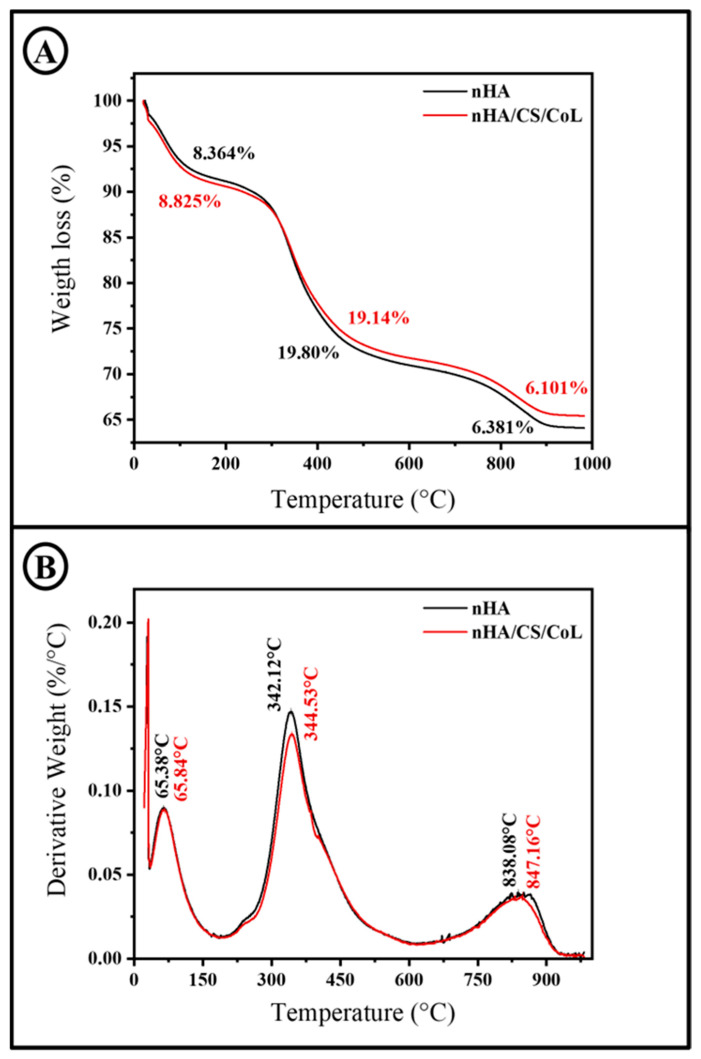
(**A**) TGA. (**B**) DTG thermograms of 3D bioprinted dental grafts.

To examine the thermal decomposition characteristics of the scaffolds, TGA and DTG curves were recorded, as illustrated in Figure 9. TGA and DTG analyses reveal that the 3D bioprinted dental graft undergoes multiple stages of weight loss upon heating.

CS and HA exhibited a gradual weight loss below 230 °C, likely due to water evaporation and dehydration within the biopolymer molecules [29]. TGA curve of nHA/CS/CoL 3D bioprinted dental graft shows a weight loss in three stages. The first stage (up to 230 °C) was associated with the loss of absorbed and bound water [56,57], with approximately a 8.83% weight loss observed in the nHA/CS/CoL sample. The second stage (230–670 °C) was related to the degradation of CS, during which approximately 20% weight loss occurred. This weight loss was attributed to the depolymerization and decomposition of the polymer chains through the deacetylation and breaking of glycosidic bonds. The third stage (670–900 °C) corresponded to the thermal degradation of the pyranose ring and the decomposition of residual carbon, resulting in a weight loss of approximately 6.10% [57]. The nHA demonstrated a weight loss in three distinct phases. The first phase, up to 210 °C, corresponded to the evaporation of adsorbed water [58], resulting in a weight reduction of approximately 8.36%. In the second phase (210–680 °C), a weight loss of about 19% was observed. The third phase (680–905 °C) showed a weight loss of approximately 6.38%. The weight losses observed in the second and third phases for both samples were attributed to the decomposition of organic components present in the raw materials [59]. Similarly, a study by Ramesh et al. demonstrated that the addition of carboxymethyl cellulose (CMC) to HA/CS scaffolds further improves thermal stability. TGA results indicated that mass degradation occurs in four temperature ranges: 25–178 °C (water loss), 178–400 °C (decomposition of CS and CMC), 400–660 °C (decomposition of HA), and 660–800 °C (decomposition of carbonate and phosphate groups in HA). The presence of CMC reinforces chemical bonding within the scaffold, thereby enhancing its thermal resistance [60]. For instance, in a study by Dalal et al., DTG curves of HA/CS composites showed three distinct peaks: the first around 97.6 °C corresponding to moisture loss, the second at approximately 304.9 °C related to the degradation of CS, and the third near 431.9 °C associated with the decomposition of HA. These findings suggest that the incorporation of HA into the CS/CoL matrix enhances the thermal stability of the scaffold by increasing the decomposition temperatures of the composite [33].

### 3.7. In Vitro Biodegradation Analysis

The temporal stability of a 3D bioprinted scaffold is a decisive factor in determining its clinical efficacy for dental bone regeneration [61]. Ideally, the degradation rate of the graft should be synchronized with the kinetics of new bone formation to ensure a seamless transition of mechanical load from the synthetic matrix to the native mineralized tissue [61]. The results of the 28-day biodegradation study in PBS (pH 7.4) at 37 °C were quantitatively summarized in Table 2. The data revealed that the composition of the bio-inks fundamentally dictates the resistance to hydrolytic degradation, with the triple-composite formulation demonstrating the most favorable profile for long-term alveolar bone support.

The degradation profiles displayed distinct behaviors across the three experimental groups. The pure nHA group exhibited a highly stable and nearly inert profile, with a cumulative weight loss of only 6.82 ± 1.12% by Day 28. This minimal mass loss is characteristic of high-purity hydroxyapatite, which possesses low solubility at physiological pH, serving as a permanent mineral template. However, for effective tissue remodeling, a degree of matrix resorption is required to allow for cellular infiltration and vascularization [62].

In contrast, the nHA/CoL group showed the highest susceptibility to degradation, reaching a mass loss of 54.78 ± 3.56% at the end of the 4-week period. The rapid degradation observed in this group, particularly after Day 14, is attributed to the high hydrophilicity of the collagenous matrix [63]. In the absence of a secondary stabilizing polymer, the collagen fibers undergo rapid swelling and subsequent hydrolytic cleavage of the peptide chains [64]. This “burst degradation” is often undesirable in dental grafting, as it can lead to premature structural failure of the alveolar ridge before sufficient biomineralization has occurred [65].

The optimized nHA/CS/CoL group demonstrated significantly controlled and balanced degradation kinetics, with a final weight loss of 25.31 ± 2.24%. By integrating CS into the nHA/CoL framework, the degradation rate was reduced by approximately 54% compared to the CS-free group. This enhanced stability is a direct result of the strong electrostatic interactions and hydrogen bonding between the cationic amino groups of chitosan and the anionic sites of the hydroxyapatite and collagen fibers [66]. This synergistic interaction creates a more compact and hydrophobic network that restricts water penetration and protects the vulnerable collagenous domains from rapid hydrolysis.

Statistical evaluation of the data in Table 2 mutes any ambiguity regarding the role of Chitosan. From Day 7 onwards, the nHA/CS/CoL group showed an extremely significant difference compared to the nHA/CoL group (*p* < 0.001, **^###^**). This level of statistical significance underscores that the stabilization provided by the triple-composite is not merely incremental but represents a fundamental structural shift. The consistency of the standard deviations (SD) across the quadruplicate measurements further confirms the homogeneity of the 3D bioprinted filaments and the reproducibility of the fabrication process.

The biodegradation behavior of the nHA/CS/CoL scaffold aligns with established findings in the field of ceramic-polymer composites. In a similar study by Lazarevic et al. [67], it was noted that the incorporation of mineral phases like hydroxyapatite into biopolymer matrices significantly enhances the chemical stability of the resulting scaffold by acting as a physical barrier against solvent diffusion. Our findings extend this principle by demonstrating that the dual-biopolymer (CS/CoL) approach provides even greater resistance than single-polymer systems.

Furthermore, the controlled mass loss of ~25% over one month is highly compatible with the physiological requirements of alveolar bone repair. As discussed by Aminatun et al. [60], a scaffold intended for bone tissue engineering must maintain its integrity for at least 3 to 6 months to support the gradual deposition of host-derived hydroxyapatite. Extrapolating the linear degradation trend observed in Table 2, the nHA/CS/CoL scaffold is projected to provide mechanical support for approximately 18–22 weeks, which perfectly matches the clinical window for dental implant loading and GBR.

The stability of the nHA/CS/CoL group also correlates with the thermal analysis results provided in Section 3.6. The high residual mass observed in the TGA profiles for the triple-composite confirms the presence of strong intermolecular bonds that resist both thermal and chemical breakdown. As reported by Mansour et al. [59], the preparation conditions and the resulting nanostructure of the hydroxyapatite phase play a vital role in the overall solubility of the biocomposite. Our results validate that the 3D bioprinting parameters used in this study successfully preserved these nanostructural advantages, yielding a graft with superior environmental resistance.

In conclusion, the quantitative and statistical evidence from the biodegradation study confirms that the nHA/CS/CoL scaffold is the most suitable candidate for dental grafting. It avoids the excessive stability of pure ceramics and the premature degradation of collagen-only systems, providing a “smart” resorption profile that is statistically validated and supported by the structural principles outlined in the contemporary biomaterials literature.

### 3.8. Cell Viability

**Figure 10:**
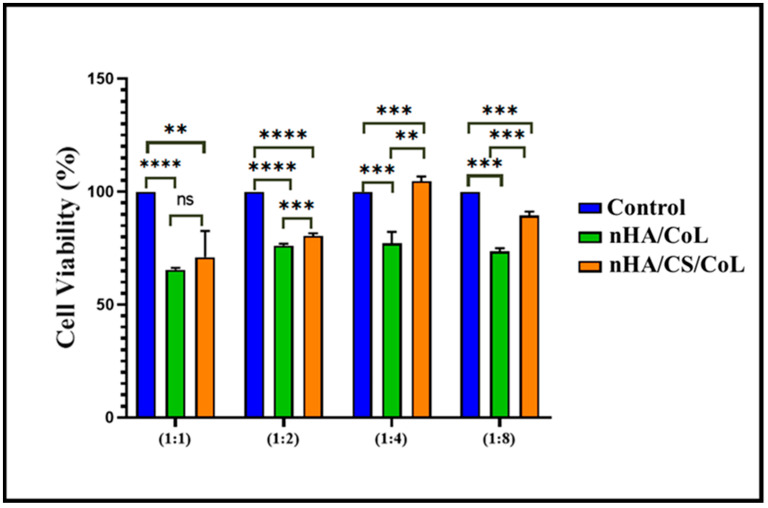
L929 cell viability values (%) of samples. Statistical significance is indicated as follows: ns = not significant (** = *p* ≤ 0.01, *** = *p* ≤ 0.001, and **** = *p* ≤ 0.0001).

An in vitro cell viability assay was conducted to assess the biocompatibility of nHA/CoL and nHA/CS/CoL using L929 mouse fibroblasts. In this study, the effects of treatments nHA/CoL and nHA/CS/CoL on cell viability were compared to the untreated control group across different concentration ratios (1:1, 1:2, 1:4, and 1:8), as shown in Figure 10. In accordance with ISO 10993-5:2009, a material is deemed non-cytotoxic if it preserves cell viability at levels exceeding 70% [68]. HA, a calcium phosphate mineral similar to the inorganic component of bone, is widely recognized for its excellent osteoconductivity and biocompatibility. Its incorporation in scaffolds has been shown to promote cell attachment and proliferation, especially for osteoblastic cell lines [69]. CS, a natural polysaccharide derived from chitin, provides a favorable environment due to its biocompatibility, biodegradability, and antimicrobial properties, further supporting cell adhesion and growth [70]. CoL, the primary structural protein in the extracellular matrix, plays a critical role in cell signaling and attachment through its integrin-binding domains, enhancing scaffold bioactivity and cell viability [71]. Our results demonstrated that the control group consistently exhibited the highest cell viability rates. Treatment with nHA/CoL showed a significant reduction in cell viability compared to control in all tested ratios (*p* < 0.0001), indicating a slight cytotoxic effect.

Conversely, treatment with nHA/CS/CoL showed a moderate decrease in cell viability compared to the control; however, this reduction was less severe than that observed with nHA/CoL treatment. Statistically significant differences between nHA/CS/CoL and control groups were found in all ratios, yet nHA/CS/CoL maintained higher viability levels compared to nHA/CoL, suggesting a relatively better cytocompatibility profile. This trend was more evident at higher dilution ratios (1:4 and 1:8), where nHA/CS/CoL’s cell viability approached closer to control values, implying a dose-dependent effect. Moreover, direct comparison between treatments nHA/CoL and nHA/CS/CoL revealed significant differences across all tested concentrations, with nHA/CS/CoL consistently supporting higher cell viability than nHA/CoL. These findings highlight that nHA/CS/CoL might be a safer alternative regarding cytotoxicity, which is critical for potential biomedical applications [40,63,72].

While the current in vitro characterization confirms the non-cytotoxic nature and high metabolic activity of L929 fibroblasts on the nHA/CS/CoL scaffolds, the absence of in vivo validation remains a limitation of this study. Alveolar bone regeneration involves complex physiological processes, including angiogenesis and immune response, which cannot be fully replicated in a 2D or 3D in vitro culture environment. Recent studies on similar calcium phosphate-biopolymer systems have demonstrated significant new bone formation and scaffold resorption in rat calvarial or alveolar defect models, supporting the regenerative potential of these material combinations [73]. The high compressive strength (~304 MPa) and favorable Ca/P ratio (2.06) of our 3D bioprinted grafts provide a strong foundation for future pre-clinical evaluations [63,74].

In this study, L929 fibroblast cells were utilized as a standardized model to confirm the non-cytotoxic nature of the 3D bioprinted dental grafts. While L929 cells provide essential data on general biocompatibility, the potential for dental bone regeneration is further supported by the presence of nHA, which serves as an osteoconductive mineral phase that facilitates cell attachment and proliferation [40]. The synergy between the mineral phase and the dual-biopolymer matrix (CS/CoL) is known to create an environment conducive to osteogenic signaling [72]. Specifically, nHA mimics the inorganic component of native bone, providing a template for biomineralization, while the RGD-like sequences in collagen support integrin-mediated cell signaling [75]. Although specific osteogenic markers such as Alkaline Phosphatase (ALP) activity and matrix mineralization were not quantified in the current preliminary study, the high metabolic activity and proliferation observed in our cell assays (Figure 10) suggest a highly bioactive microenvironment. Future studies involving MC3T3-E1 or Saos-2 osteoblastic lines will be conducted to further delineate the molecular mechanisms of bone-scaffold integration and long-term mineralized tissue formation.

### 3.9. Clinical Challenges and Future Perspectives

While the nHA/CS/CoL scaffolds demonstrate superior mechanical and biological performance in vitro, several challenges must be addressed to ensure successful clinical translation into dental practice. The transition from a laboratory-scale 3D bioprinting process to a standardized clinical product involves overcoming hurdles in sterilization, scalability, and long-term biological control.

#### 3.9.1. Sterilization and Material Integrity

A primary challenge is the sterilization of biopolymer-ceramic composites without compromising their structural or biological properties. Standard autoclaving is unsuitable for the nHA/CS/CoL scaffolds, as high temperatures and moisture can trigger the premature degradation of collagen and alter the crystallinity of nanohydroxyapatite [76]. In this study, we utilized UV-C irradiation as a preliminary sterilization method, but for clinical use, low-temperature sterilization techniques such as ethylene oxide (EtO) or gamma irradiation must be optimized [77]. However, these methods can induce cross-linking or chain scission in chitosan and collagen, potentially altering the controlled biodegradation rate of 25.3% per month [78,79] that we have established in our 28-day study.

#### 3.9.2. Scalability of Patient-Specific CAD

The core innovation of this study lies in the use of extrusion-based 3D bioprinting to create patient-specific dental constructs. However, the clinical workflow from CBCT imaging to CAD modeling and final printing requires seamless integration [80]. Current limitations include the resolution of CAD-to-print translation for intricate alveolar defects and the speed of fabrication. While our rheological data confirms high thixotropic recovery, maintaining the precision of complex geometries during large-scale production remains a technical challenge. Future work must focus on automated algorithms that can translate patient-specific defect geometries into optimized toolpaths for our nHA-reinforced bio-inks.

#### 3.9.3. In Vivo Degradation and Host Response

As highlighted by our in vitro results, the nHA/CS/CoL group provides a statistically significant improvement in stability over CS-free groups. However, the oral environment is dynamic, characterized by varying pH levels and enzymatic activity (lysozyme and collagenase) that can accelerate scaffold resorption. A critical future perspective is the synchronization of this resorption with the patient’s individual bone remodeling rate. If the scaffold degrades too rapidly, the mechanical support for a dental implant may be lost; if it degrades too slowly, it may hinder the formation of dense, mineralized alveolar bone [81,82].

To move beyond the limitations of this characterization, the next phase of this research will involve the following:

- Osteogenic Differentiation: Utilizing MC3T3-E1 osteoblastic lines to quantify mineralization and ALP activity.
- Animal Models: Validating the regenerative capacity in rat alveolar bone defect models to observe the immune response and vascularization.
- Bio-ink Functionalization: Incorporating growth factors or antimicrobial agents to further enhance the healing microenvironment.

By addressing these clinical challenges, the 3D bioprinted nHA/CS/CoL grafts developed in this study can evolve into a highly effective, personalized regenerative therapy, bridging the gap between advanced biomaterials science and chairside dental surgery.

## 4. Conclusions

In this study, HA-reinforced CS/CoL 3D bioprinted dental grafts were successfully developed using 3D bioprinting technology for potential use in dental tissue engineering. The structural, mechanical, and biological evaluations confirmed that the designed scaffolds exhibit favorable characteristics for supporting alveolar bone regeneration and gingival tissue integration. The use of biocompatible and biodegradable materials, combined with the ability to fabricate patient-specific architectures, presents a significant advancement over conventional grafting approaches. The composite scaffolds containing HA, CS, and CoL evaluated in this study demonstrate promising potential for use as 3D bioprinted dental grafts due to their favorable thermal stability, biocompatibility, and structural integrity. TGA and DTG analyses revealed that these materials exhibit thermal stability across different temperature ranges and possess a high water retention capacity. These findings suggest that the 3D bioprinted dental grafts may withstand sterilization and physiological conditions encountered in the oral environment. Accordingly, 3D bioprinted dental grafts may serve as effective biomaterial candidates, particularly for bone regeneration and periodontal tissue engineering applications in the dental field.

Furthermore, the demonstrated cell compatibility highlights the potential of these 3D bioprinted dental grafts to enhance tissue regeneration and healing in clinical applications. Overall, this study contributes to the growing body of research focused on personalized and functional biomaterials for dental regenerative therapies and underscores the promise of 3D bioprinting in producing next-generation scaffolds tailored to individual patient needs. Although the regenerative capacity was assessed through rigorous in vitro characterization and mechanical benchmarking, future in vivo studies using animal defect models are required to confirm the long-term integration and bone-remodeling potential of the nHA/CS/CoL scaffolds in a dynamic biological environment.

## Figures and Tables

**Table 1:** Mechanical properties of 3D bioprinted dental grafts (*n* = 4).

Sample Group	Max. Compressive Strength (MPa)	Elastic Modulus (MPa)	Strain at Failure (%)
nHA	239.12 ± 12.45	7540 ± 155.3	3.18 ± 0.14
nHA/CoL	304.56 ± 14.82 ***^,##^	8192 ± 182.1 **^,#^	4.12 ± 0.22 ***
nHA/CS/CoL	198.45 ± 10.30	7795 ± 164.8	2.55 ± 0.11

Notes: **, *** indicate significant difference compared to nHA group (*p* < 0.01, *p* < 0.001, respectively). **^#, ##^** indicate significant difference compared to nHA/CoL group (*p* < 0.05, *p* < 0.01).

**Table 2:** Cumulative weight loss (%) of bioprinted scaffolds over 28 days in PBS (*n* = 3).

Days	nHA (%)	nHA/CoL (%)	nHA/CS/CoL (%)
Day 1	1.25 ± 0.32	4.52 ± 0.81	2.14 ± 0.54
Day 7	2.84 ± 0.56	15.21 ± 1.24	8.45 ± 0.92 ***^,##^
Day 14	4.18 ± 0.72	28.45 ± 2.18	14.22 ± 1.45 ***^,###^
Day 21	5.56 ± 0.88	42.12 ± 2.84	19.54 ± 1.82 ***^,###^
Day 28	6.82 ± 1.12	54.78 ± 3.56	25.31 ± 2.24 ***^,###^

Notes: Values are expressed as ± SD. *** indicate significant difference compared to the nHA group (*p* < 0.001). **^##^,** ^###^ indicate significant difference compared to the nHA/CoL group (*p* < 0.01, *p* < 0.001).

## Data Availability

The original contributions presented in this study are included in the article. Further inquiries can be directed to the corresponding authors.
